# Molecular chaperones: guardians of the proteome in normal and disease states

**DOI:** 10.12688/f1000research.7214.1

**Published:** 2015-12-15

**Authors:** Wilson Jeng, Sukyeong Lee, Nuri Sung, Jungsoon Lee, Francis T.F. Tsai

**Affiliations:** 1 Verna and Marrs McLean Department of Biochemistry and Molecular Biology, Baylor College of Medicine, Houston, TX, USA; 2 Department of Molecular and Cellular Biology, Baylor College of Medicine, Houston, TX, USA; 3 Department of Molecular Virology and Microbiology, Baylor College of Medicine, Houston, TX, USA

**Keywords:** molecular chaperones, chaperones, proteases, protein folding, misfolding, aggregation, ATP-dependent molecular chaperones

## Abstract

Proteins must adopt a defined three-dimensional structure in order to gain functional activity, or must they? An ever-increasing number of intrinsically disordered proteins and amyloid-forming polypeptides challenge this dogma. While molecular chaperones and proteases are traditionally associated with protein quality control inside the cell, it is now apparent that molecular chaperones not only promote protein folding in the “forward” direction by facilitating folding and preventing misfolding and aggregation, but also facilitate protein unfolding and even disaggregation resulting in the recovery of functional protein from aggregates. Here, we review our current understanding of ATP-dependent molecular chaperones that harness the energy of ATP binding and hydrolysis to fuel their chaperone functions. An emerging theme is that most of these chaperones do not work alone, but instead function together with other chaperone systems to maintain the proteome. Hence, molecular chaperones are the major component of the proteostasis network that guards and protects the proteome from damage. Furthermore, while a decline of this network is detrimental to cell and organismal health, a controlled perturbation of the proteostasis network may offer new therapeutic avenues against human diseases.

## Introduction

The vast majority of proteins must fold correctly in order to gain functional activity. While the protein folding information is encoded within the nascent polypeptide chain, newly synthesized polypeptides (or those imported into organelles) are prone to misfolding, causing aggregation and formation of other toxic species ^1^. Consequently, maintaining protein homeostasis (proteostasis) is essential for cell and organismal health ^2^. To accomplish this, cells have evolved a sophisticated network of protein quality control machines, consisting of molecular chaperones and proteases, which monitor the folding of proteins and their assembly into functional complexes, and selectively remove excess and damaged proteins from the cell. Challenging the capacity of this proteostasis network increases the risk of human diseases associated with protein misfolding and aggregation ^1^.

While most proteins adopt a defined three-dimensional structure, several exceptions are known to exist. Notable examples include prions that can adopt multiple, distinct, three-dimensional structures ^3– 5^, and an ever-increasing number of intrinsically disordered proteins (IDPs), which feature large regions of random coil or lack a defined structure altogether ^6– 8^. At least in yeast, it is now widely accepted that molecular chaperones play an essential role in prion replication ^9, 10^ by governing the inheritance and maintenance of yeast prions, and in some cases their elimination by chaperone overexpression ^11– 15^. However, concrete evidence of an involvement of molecular chaperones in mammalian prion replication, although proposed ^16^, is missing, and whether molecular chaperones play a role in the stabilization and/or protection of IDPs remains uncertain.

What is a molecular chaperone? A molecular chaperone can be generally defined as any protein that assists other macromolecules in folding and/or assembling into higher order structures, without it being a component of these final structures ^17^. Thus, while their main function inside the cell is to assist in the folding and maturation of unfolded or partially folded macromolecules and to prevent their misfolding and aggregation, it was widely assumed that molecular chaperones involved in *de novo* protein folding do not recover functional protein once aggregation has occurred. This concept was challenged by the discovery of a novel stress-inducible molecular chaperone known as Hsp104 ^18^, which functions as an ATP-dependent protein disaggregase that rescues stress-damaged proteins from a previously aggregated state ^19, 20^. The discovery of Hsp104 has since expanded our definition of molecular chaperones to include those that promote the forward folding or prevent the aggregation of proteins on one hand, and those that recover functional protein from aggregates on the other hand.

At the molecular level, molecular chaperones come in diverse shapes and sizes, and can be broadly separated into two groups: those that depend on metabolic energy to fuel their chaperone activity, and those that do not ^21^. Examples of the former include all ATP-dependent molecular chaperones ^22^, while the latter include small heat shock proteins ^23^, protein disulfide isomerase ^24^, ribosome-associated chaperones such as trigger factor ^25^, and conditionally activated chaperones ^26^.

**Figure 1.**
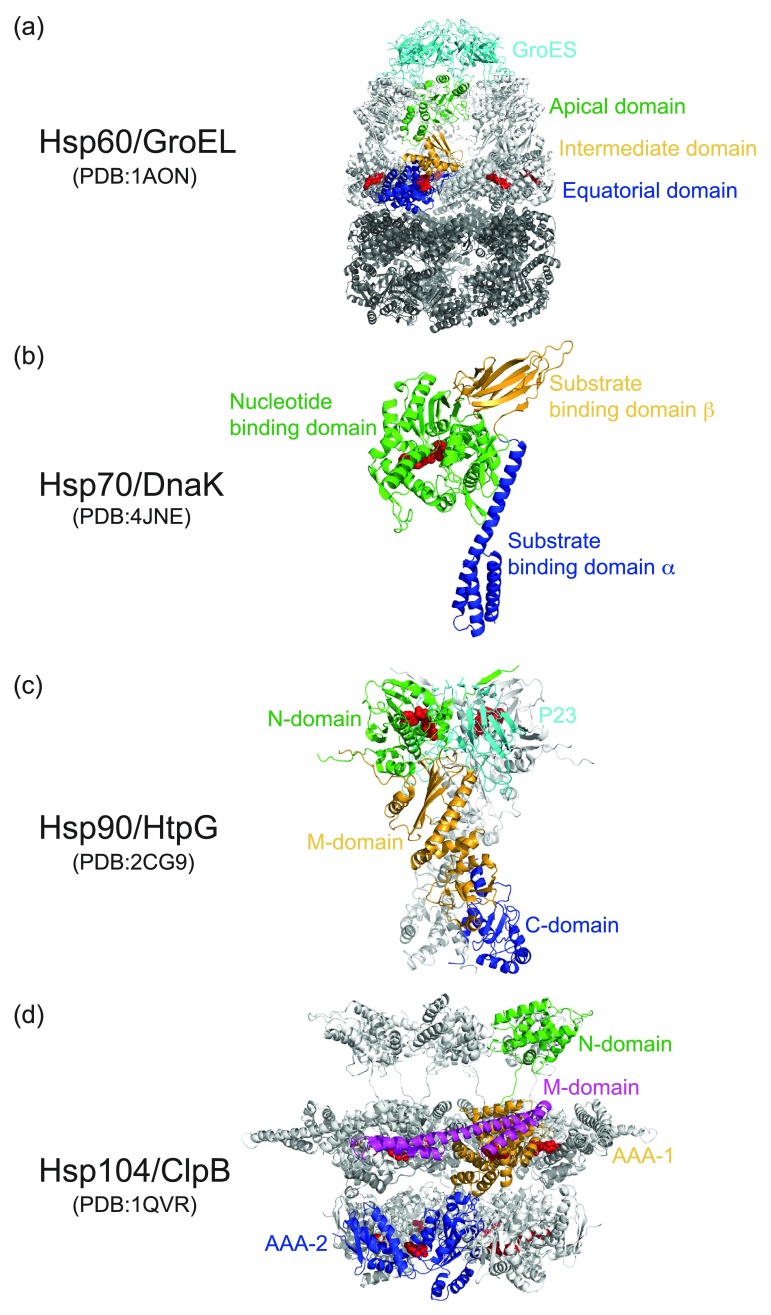
Molecular architecture and domain organization of ATP-dependent molecular chaperones. Protein is shown as ribbon diagram with the bound nucleotide as red CPK model. For each chaperone, the domains of one subunit are shown in different colors in order of green, orange, and blue from N- to C-termini. Bound co-chaperones are colored cyan. ( **a**) Hsp60/GroEL: Architecture and domain organization of the *E. coli* GroEL tetradecamer bound to ADP with a GroES heptamer capping the GroEL *cis* ring (PDB: 1AON) ^33^. ( **b**) Hsp70/DnaK: Architecture and domain organization of the *E. coli* DnaK monomer in the ATP-bound state (PDB: 4JNE) ^54^. ( **c**) Hsp90/HtpG: Architecture and domain organization of the ATP-bound yeast Hsp90 dimer in the closed-state conformation, and its stabilization by p23/Sba1 (PDB: 2CG9) ^81^. ( **d**) Hsp104/ClpB: Architecture and domain organization of a yeast Hsp104 hexamer bound to ATP (PDB: 1QVR; EMD-1631) ^97, 99^. The Hsp104 M-domain that mediates the species-specific interaction with Hsp70 is colored in magenta.

The focus of this review is on ATP-dependent molecular chaperones that harness the energy from ATP binding and/or hydrolysis to assist protein folding and unfolding (i.e., disaggregation). Their cellular expression can be either constitutive in order to perform vital housekeeping functions, or inducible by short exposure to elevated temperatures or other forms of stress that cause protein denaturation. Those that are stress-inducible are also known as heat-shock proteins or HSPs, while those that are constitutively active are termed heat-shock cognates or HSCs. Different members of both groups are classified according to their molecular weight, for example, HSP of 60-kDa (Hsp60), 70-kDa (Hsp70), 90-kDa (Hsp90), and 100-kDa (Hsp100), although many are better known by their common name that is used to designate each chaperone homolog from eubacteria, for example, GroEL (Hsp60), DnaK (Hsp70), HtpG (Hsp90), and ClpB (Hsp100) ( Figure 1). All of these aforementioned HSPs bind adenine nucleotide and hydrolyze ATP. Furthermore, another common feature is their cooperation with other proteins, termed co-chaperones, which regulate the ATPase and/or chaperone activity, or reset the functional cycle.

## The Hsp60 family

Hsp60 chaperones are known as chaperonins ^27^, and can be divided into two subgroups. Group I chaperonins are sevenfold symmetric and assemble into a barrel-like structure composed of two rings of seven identical subunits ^28^. Notable examples include bacterial GroEL and Hsp60 from mitochondria and chloroplasts. Each GroEL subunit consists of an equatorial, intermediate, and apical domain ( Figure 1a) ^29– 31^. ATP binding triggers a conformational rearrangement of the apical domains followed by GroES binding. The latter is a GroEL co-chaperone that assembles into a heptamer ring ^32^, and caps one side of the GroEL barrel (the *cis* ring) ^33^ to encapsulate the substrate ^34^, and to promote protein folding ^35^. The prevailing model suggests that GroEL-ES promotes folding through repetitive binding, encapsulation, and release of the substrate protein ^28, 36^. Group II chaperonins are homo- or hetero-oligomers forming an eightfold double barrel structure composed of sixteen subunits, and include the eukaryotic chaperonin containing TCP1 complex (CCT), also known as the TCP-1 Ring Complex (TRiC), and the thermosome and *Methanococcus maripaludis* chaperonin (Mm-Cpn) from Archaea ^37^. Unlike Group I chaperonins, group II members do not function together with a GroES-like co-chaperone, but instead contain a built-in lid that undergoes an iris-like motion to promote protein folding ^38^.

Much of our current understanding of chaperonin function comes from seminal work on *Escherichia coli* GroEL. *E. coli* GroEL is essential since many vital proteins, including metabolic enzymes and components of the transcription-translation machinery, depend on the GroE system for folding ^39^. While GroEL’s essential housekeeping function is beginning to be understood, an emerging question is the recent appreciation of multiple copies of GroEL in some bacterial genomes ^40^, as seen in actinobacteria, which includes *Mycobacterium tuberculosis*, the causative agent of Tuberculosis (TB). TB accounts for ~2 million deaths annually and is a major public health problem exacerbated by the emergence and rapid spread of new multidrug-resistant *M. tuberculosis* strains. *M. tuberculosis* encodes two copies of *groEL* in its genome ^40^. While *M. tuberculosis* GroEL2 is essential for viability, the function of the non-essential GroEL1 paralog remains less clear. The crystal structures of *M. tuberculosis* GroEL2 and a GroEL1 fragment showed that the apical domains have nearly identical three-dimensional structures ^41, 42^. While GroEL2 is believed to be the housekeeping chaperonin similar to *E. coli* GroEL, GroEL1 may function as a specialized chaperonin with a more limited substrate spectrum. Consistent with this notion, it has been proposed that mycobacterial GroEL1 is a dedicated chaperone for biofilm formation ^43^, which is presumed to confer the extraordinary starvation survival and resistance of *M. tuberculosis* to known antibiotics ^44^.

## The Hsp70 family

Members of the Hsp70 chaperone family are found in all three surviving domains of life ^45^. At the molecular level, Hsp70 is a two-domain protein consisting of a nucleotide-binding domain connected by a long and flexible hydrophobic linker to the substrate-binding domain that can be subdivided into a β-sandwich domain and an α-helical domain ( Figure 1b). Furthermore, cytosolic eukaryotic Hsp70s feature a Glu-Glu-Val-Asp or “EEVD” motif at the extreme C-terminus, which is required for the interaction with Hsp70 co-chaperones that regulate the Hsp70 ATPase activity and its ability to bind substrate ^46^. It has been shown that bacterial Hsp70 recognizes diverse polypeptides mostly in an unfolded or partially unfolded form by binding to a four to five residue stretch of hydrophobic amino acids flanked by regions enriched in basic amino acids ^47^, which occur on average every 30–40 residues in most proteins. Since Hsp70 binding motifs are typically buried within the correctly folded protein, it provides a means to selectively seek out and bind proteins that are in a non-native conformation. However, Hsp70 chaperones rarely, if ever, function on their own and require the assistance of co-chaperones, which include nucleotide exchange factors, such as bacterial GrpE and eukaryotic Hsp110, and the large family of J-domain-containing Hsp40 co-chaperones, which accelerate ATP hydrolysis, serve as substrate targeting factors, and stabilize Hsp70-substrate interaction ^21^.

Over the last decade, high-resolution structural information on full-length Hsp70 chaperones has become available ^48– 54^, providing new insight into the Hsp70 conformational cycle and its allosteric regulation by nucleotide ^55^. Hsp70 function is controlled by nucleotide binding with ATP, promoting an open-conformation with low substrate-binding affinity, and ADP, promoting a closed-conformation required for tight binding of substrates ^21^.

In addition to Hsp70’s known role in protein folding, Hsp70 also has other non-chaperone functions. For instance, it was recently shown that Hsp70 functions as an activator of the ring-forming Hsp104 protein disaggregase and is required to unleash the potent protein disaggregating activity ^56, 57^. While no Hsp104 homolog is known to exist in metazoans, the discovery of a mammalian protein disaggregase, composed of Hsp70, Hsp110, and Hsp40, is exciting and supports functional conservation of a protein disaggregating activity in animal cells ^58– 61^. However, despite its nomenclature, Hsp110 is not an Hsp100 homolog, but instead belongs to an Hsp70 subfamily that is activated by nucleotide ^62^, shares structural ^49, 63, 64^ and perhaps functional conservation with Hsp70 ^65^, and functions as an Hsp70 nucleotide exchange factor ^66, 67^.

## The Hsp90 family

Hsp90 belongs to a conserved group of ATP-dependent molecular chaperones ^68– 70^ which, together with Hsp70 and a cohort of co-chaperones, facilitates the late-stage folding and maturation of proteins ^71, 72^. Since Hsp90 substrates are mostly substantially folded proteins, they are known as “client proteins” ^68^ to distinguish them from other chaperone substrates that lack a defined structure. More than 400 different clients are known to depend on Hsp90 for folding or maturation, and include protein kinases, transcription factors, and E3 ubiquitin ligases ^73^. The large number of signaling and tumor promoting proteins amongst Hsp90 clients has made Hsp90 a promising drug target ^74^.

Apart from Hsp90 chaperones in the eukaryotic cytosol, Hsp90 homologs are found in bacteria (HtpG) and eukaryotic organelles, including the endoplasmic reticulum (Grp94), mitochondrion (TRAP1), and chloroplast ^75^. Interestingly, Hsp90-like domains with chaperone activity have also been found in Sacsin, a 521-kDa protein associated with an autosomal recessive form of spastic ataxia ^76, 77^. However, an Hsp90 homolog has not been found in Archaea.

Hsp90 chaperones share a similar domain structure consisting of an N-terminal (N-) nucleotide-binding domain, a middle (M-) domain, and a C-terminal (C-) dimerization domain ( Figure 1c). The N-domain is connected to the M-domain *via* a flexible linker that is often highly charged and, in human Hsp90, is over 60 residues in length. While important to cytosolic eukaryotic Hsp90 function ^78– 80^, the charged-linker is not universally conserved and is essentially absent in both bacterial and mitochondrial Hsp90s. Crystal structures are now available for full-length members of all Hsp90 subfamilies mostly with bound nucleotide ^81– 84^, including the recent structure of an asymmetric TRAP1 dimer in the ATP-bound state ^84^. The latter lends supports for a sequential ATP hydrolysis mechanism ^85, 86^, although asymmetric binding of nucleotide was not observed ^84^. Consistent with the prevailing notion, the available structures confirmed that all Hsp90 chaperones form homodimers with the N-domain mediating nucleotide binding. Strikingly, however, apo Hsp90 forms a wide-open, V-shaped dimer with the N-domains separated by over 100 Å ^82^, while Hsp90 in the ATP-bound state adopts an intertwined, N-terminally closed dimer ^81, 84^. Since the N-domains are too far apart in the open-state to signal the nucleotide status between neighboring subunits, how ATP-binding induces the closed-state conformation remains an open question. One model suggests that Hsp90 chaperones sample different three-dimensional conformations with different adenine nucleotides stabilizing distinct Hsp90 dimer conformations ^87– 89^. While not mutually exclusive, the crystal structures of intact Grp94, which were determined in the ATP- and ADP-bound state, revealed a very similar Hsp90 dimer conformation irrespective of the nature of the bound nucleotide ^83^. Hence, further *in vitro* and *in vivo* studies are needed to address the exact roles of ATP and ADP for Hsp90 chaperone function.

## The Hsp100 family

Members of the Hsp100 family were first discovered as the protein-activated ATPase components of the protease Ti from *E. coli* ^90, 91^, now better known as the ClpAP protease. Members of the Hsp100/Clp family belong to the large superfamily of ATPases Associated with diverse cellular Activities (AAA+) ^92, 93^. Hsp100/Clp members form a hexameric ring structure and function as the protein-unfolding component of chambered proteases ^94, 95^. The discovery of yeast Hsp104 that facilitates protein disaggregation ^19^, as opposed to targeting proteins for degradation, established Hsp104 as the founding member of a new family of ATP-dependent molecular chaperones. In addition to yeast Hsp104, Hsp104 homologs were found subsequently in bacteria (ClpB), plants (Hsp101), and most recently in *Dictyostelium discoideum* (Hsp101) ^96^.

Like all Hsp100/Clp proteins, Hsp104 forms an oligomer, with the homohexamer being the functionally active form ^97– 100^. Hsp104 features two canonical Walker-type ATP-binding domains, known as AAA domains, in addition to several other structural elements that define members of the AAA+ superfamily and include the so-called arginine-finger and the sensor 1 and 2 motifs ^101– 106^. While the Hsp104 hexamer is stabilized by nucleotide and is an active ATPase *in vitro* ^107, 108^, it requires the cooperation of the cognate Hsp70 chaperone system, consisting of Hsp70 and Hsp40 in yeast ^20^ and DnaK, DnaJ, and GrpE in eubacteria ^109– 111^, to recover functional protein from aggregates.

At the molecular level, Hsp104 consists of an N-terminal domain, and two tandem AAA+ domains, termed AAA-1 and AAA-2 ( Figure 1d) ^97, 103^. The AAA-1 domain features an 85-Å long coiled-coil insertion, known as the M-domain, which is located on the outside of the hexamer ^99, 100, 112^ and distinguishes Hsp104 members from other Hsp100/Clp ATPases. The M-domain is essential for protein disaggregation by mediating the interaction between Hsp104 and Hsp70 ^113– 115^, and may function as a molecular toggle to allosterically control the ATPase and mechanical activities of the Hsp104 motor ^116^.

How Hsp104 facilitates protein disaggregation has been revealed by the combined efforts of several groups ^117^. It is now widely accepted that, inside the cell, the Hsp70 system targets the Hsp104 motor to both amorphous and ordered aggregates ^15, 118^, from which Hsp104 extracts polypeptides using an ATP-driven power stroke involving pore loops present in the AAA-1 and AAA-2 domains ^119^, and threading the polypeptide through the central channel of the Hsp104 hexamer ^120, 121^.

While we are beginning to understand the function of the M- and AAA domains, the role of the N-domain is less clear. It was shown that the N-domain is dispensable for Hsp104 function *in vitro* and *in vivo* ^15, 103, 122– 125^. However, others found that the N-domain is essential for bacterial Hsp104 ^126, 127^ and mediates substrate interaction ^126, 128– 131^. Consistently, the N-domain of yeast Hsp104 enhances protein disaggregation *in vitro* ^114^, mediates prion interaction in yeast ^132^, and is essential for yeast prion dissolution ^112^ and curing by Hsp104 overexpression ^124^.

In addition to Hsp104’s role in yeast stress responses and yeast prion replication, new roles are emerging, including the asymmetric distribution of oxidative damaged proteins ^133, 134^, facilitating the sorting of tail-anchored proteins to the endoplasmic reticulum membrane ^135^, and septin folding and assembly ^136^. Hence, future studies will provide a more complete picture as to the extent of Hsp104’s cellular function.

## Future perspectives

It is now widely appreciated that molecular chaperones are intimately linked to proteostasis maintenance and are essential to cell and organismal health. Perturbation of the proteostasis network, for instance by “chaperone overload” ^137^ or polyglutamine expansion ^138^, invariably disrupts the balance of the protein folding landscape triggering protein misfolding and the formation of aggregates that are hallmarks of neurodegenerative diseases, prion-mediated infections, and amyloidosis. At the same time, a controlled perturbation of the functional interaction between molecular chaperones and proteases could provide new avenues for therapeutic intervention. This could be achieved by using small molecule compounds, or by RNA interference, or restoring the proteostasis network in disease states, for instance with chemical chaperones or by induced chaperone expression ^139, 140^.
